# Genome wide transcriptome profiling of a murine acute melioidosis model reveals new insights into how *Burkholderia pseudomallei *overcomes host innate immunity

**DOI:** 10.1186/1471-2164-11-672

**Published:** 2010-11-27

**Authors:** Chui-Yoke Chin, Denise M Monack, Sheila Nathan

**Affiliations:** 1 School of Biosciences and Biotechnology, Faculty of Science and Technology, Universiti Kebangsaan Malaysia, 43600 UKM Bangi Selangor D. E. Malaysia; 2 Department of Microbiology and Immunology, Stanford University School of Medicine, Stanford, CA 94305-5120, USA; 3 Malaysia Genome Institute, UKM-MTDC Technology Centre, 43600 UKM Bangi, Selangor D. E. Malaysia

## Abstract

**Background:**

At present, very little is known about how *Burkholderia pseudomallei *(*B. pseudomallei*) interacts with its host to elicit melioidosis symptoms. We established a murine acute-phase melioidosis model and used DNA microarray technology to investigate the global host/pathogen interaction. We compared the transcriptome of infected liver and spleen with uninfected tissues over an infection period of 42 hr to identify genes whose expression is altered in response to an acute infection.

**Results:**

Viable *B. pseudomallei *cells were consistently detected in the blood, liver and spleen during the 42 hr course of infection. Microarray analysis of the liver and spleen over this time course demonstrated that genes involved in immune response, stress response, cell cycle regulation, proteasomal degradation, cellular metabolism and signal transduction pathways were differentially regulated. Up regulation of toll-like receptor 2 (TLR2) gene expression suggested that a TLR2-mediated signalling pathway is responsible for recognition and initiation of an inflammatory response to the acute *B. pseudomallei *infection. Most of the highly elevated inflammatory genes are a cohort of "core host immune response" genes commonly seen in general inflammation infections. Concomitant to this initial inflammatory response, we observed an increase in transcripts associated with cell-death, caspase activation and peptidoglysis that ultimately promote tissue injury in the host. The complement system responsible for restoring host cellular homeostasis and eliminating intracellular bacteria was activated only after 24 hr post-infection. However, at this time point, diverse host nutrient metabolic and cellular pathways including glycolysis, fatty acid metabolism and tricarboxylic acid (TCA) cycle were repressed.

**Conclusions:**

This detailed picture of the host transcriptional response during acute melioidosis highlights a broad range of innate immune mechanisms that are activated in the host within 24 hrs, including the core immune response commonly seen in general inflammatory infections. Nevertheless, this activation is suppressed at 42 hr post-infection and in addition, suboptimal activation and function of the downstream complement system promotes uncontrolled spread of the bacteria.

## Background

How organisms respond appropriately to *B. pseudomallei*, the causative agent of melioidosis, remains a central question within the Burkholderia community. Over the past decade, knowledge on the pathogenesis of *B. pseudomallei *has increased considerably. However, very little is known about the molecular mechanisms that underlie *B. pseudomallei *virulence and how this organism is able to interact with its host to elicit melioidosis symptoms. Melioidosis can present with an array of clinical symptoms. Clinically apparent infections range from acute or chronic localized infection involving a single organ, to fulminant septicaemia in multiple organs (liver, spleen, lung and prostate) and septic shock [1]. The disease may become dormant and the infected person may relapse after months, years or decades (the longest recorded incubation period documented is 62 years) [2]. The factors influencing disease outcome are not known, although it has been suggested that differences in the virulence of different infecting strains, the route of inoculation and inoculum size might contribute to the clinical outcome of disease [3]. Underlying diabetes mellitus and chronic renal failure are major predisposing factors of melioidosis [2-5]. Recently, the risk factor was extended to individuals who were uninjured bystanders during the tsunami of December 2004 [6].

BALB/c mice infected with *B. pseudomallei *die of septicemic disease with overwhelming bacterial loads in organs and blood, accompanied by organ inflammation and necrosis a few days after infection, reflecting a failure of the host innate immune response [7]. mRNA for proinflammatory cytokines such as tumor necrosis factor (TNF) -α, interferon (IFN) -γ and interleukin (IL) -6 (IL6) were detected earlier and in more abundance in the organs of intravenously infected BALB/c mice with acute disease compared to the more resistant C57BL/6 mice [8]. Additionally, Santanirand et al. [9] reported that an early control mechanism is dependent upon the rapid production of IFN- γ, because IFN- γ primes macrophages to increase their bactericidal activity towards *B. pseudomallei*. Gan (2005) reported that the development of acute disease is not due to a lack of but rather an excess of inflammation, reflecting a failure of regulatory mechanisms. Melioidosis patients also exhibit elevated serum levels of pro-inflammatory cytokines such as IFN-γ, TNF-α, chemokine (C-X-C motif) ligand 9 (MIG) and chemokine (C-X-C motif) ligand 10 (IP10) [10].

In recent years, many studies have focused on the general immune response to shed light on the *B. pseudomallei*-host interaction. However, to date, a full and complete picture of host responses to this pathogen is still not available. The purpose of this study was to develop a comprehensive picture of the host transcriptional response during the acute stage of melioidosis. Insight into the events at the early infection stage will improve our understanding of the immediate host responses to counteract this pathogen. To address this, we developed systemic acute melioidosis infection of mice and performed transcriptional analysis of the liver and spleen isolated from mice infected over a 42 hr time period. Our analysis identified several thousand genes whose expression was altered in *B. pseudomallei*-infected mice. Most notably, the majority of the identified genes were involved in immune response, stress response, cell cycle regulation, proteasomal degradation, cellular metabolism and signal transduction pathways. At the early phase of infection, most of the differentially expressed genes are those involved in the immediate immune responses. However, at 24 hr post-infection (hpi), the majority of the genes were involved in host cellular metabolism and signal transduction pathways and found to be down-regulated. These results suggest that numerous cellular processes were transcriptionally altered throughout the course of the host response to *B. pseudomallei*.

## Results

### Development and characterization of acute melioidosis in a mouse model

BALB/c mice were challenged with three *B. pseudomallei *local clinical isolates (referred to herein as D286, H10 and R15) via the intravenous (i.v.) route. The ten-day LD_50 _was determined for each isolate as shown in Additional file 1, Figure S1. The 10-day LD_50 _for *B. pseudomallei *D286, H10 and R15 are 5.55 × 10^2 ^CFU, 5.63 × 10^3 ^CFU and 2.2 × 10^5 ^CFU, respectively. The mice infected with a dosage of >10^4 ^CFU *B. pseudomallei *D286 were lethargic, had ruffled fur and developed paresis of both hind legs at the late stage of the course of infection ultimately leading to paralysis before succumbing to infection, similar to a previous report [11]. Based on the lower LD_50 _value, the D286 isolate was chosen for the following experiments.

**Figure 1:**
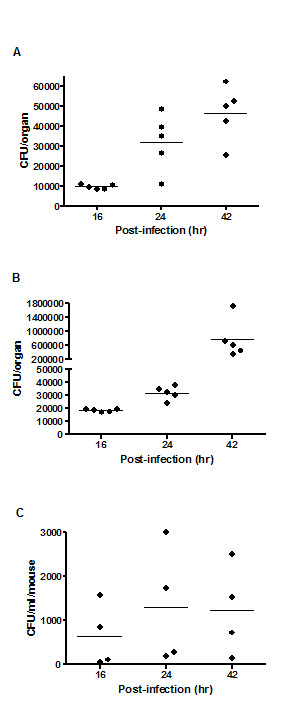
**Bacterial loads in BALB/c mice**. The bacterial loads in the (**A**) liver, (**B**) spleen and (**C**) blood of BALB/c mice at 16 hr, 24 hr and 42 hr time points after intravenous infection with 1.1 × 10^3 ^CFU of *B. pseudomallei *D286 are shown. Each symbol represents one mouse. Horizontal line indicates the geometric mean for each group. The control mice are not represented as no colonies grew from their organ homogenates or blood samples.

To characterize the acute melioidosis model, we monitored the kinetics of the bacterial loads in various organs and leukocyte differential counts during the course of infection in BALB/c mice infected with 1.1 × 10^3 ^CFU of *B. pseudomallei *D286. At 16 hpi, the bacterial load in the spleen (10^4 ^CFU/organ) was significantly higher than the liver (10^3 ^CFU/organ) (p-value = 0.0009) while the bacterial load in both organs were similar at 24 hpi and 42 hpi, with an average of 10^4 ^to 10^5 ^CFU/organ (Figure 1). The data demonstrates that no significant differences exist in bacterial replication and dissemination within these two organs during the first 42 hr of infection. During the course of infection, viable *B. pseudomallei *were also detected in the blood, although at lower numbers (~10^2 ^CFU/ml). High numbers of *B. pseudomallei *in various organs, as well as presence in the blood confirms that systemic acute septicemic melioidosis was successfully developed in BALB/c mice. No significant differences were observed in liver and spleen weights at all infection time points (data not shown) and no clinical signs of illness were observed when compared to the naïve mice.

**Figure 2:**
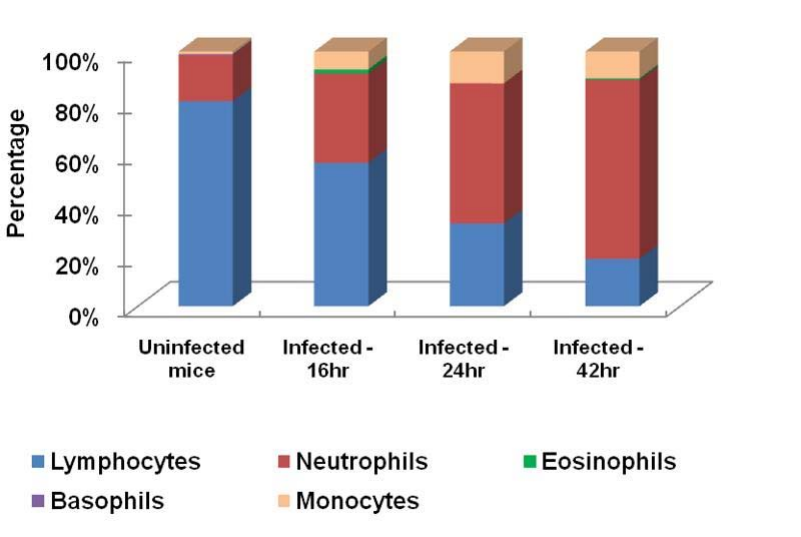
**Leukocyte differential counts during acute *B. pseudomallei *infection**. Changes in differential leukocyte counts after intravenous infection with 1.1 × 10^3 ^CFU *B. pseudomallei *D286. Values are given as that of pooled-blood from infected mice for each group at the particular time point.

To determine changes in leukocyte counts and composition during infection of BALB/c mice, blood samples from 16, 24 and 42 hr time points were analyzed. The results of the differential blood film after infection with 1.1 × 10^3 ^CFU *B. pseudomallei *D286 revealed a rise in the number of neutrophils over the course of infection (Figure 2). In addition, a decline in lymphocyte counts and finally development of lymphopenia was observed in these *B. pseudomallei *infected mice as reported previously [5]. The neutrophil counts increased about 4-fold as compared to the naïve mice and the size and shape of erythrocytes and leukocytes were normal (data not shown), demonstrating that haematopoiesis of the host was not affected by the bacteria during the course of infection. The rise in number of granulocytes indicates the innate immune mechanism was triggered in response to *B. pseudomallei *infection.

### Global transcriptional responses to acute stage melioidosis

**Figure 3:**
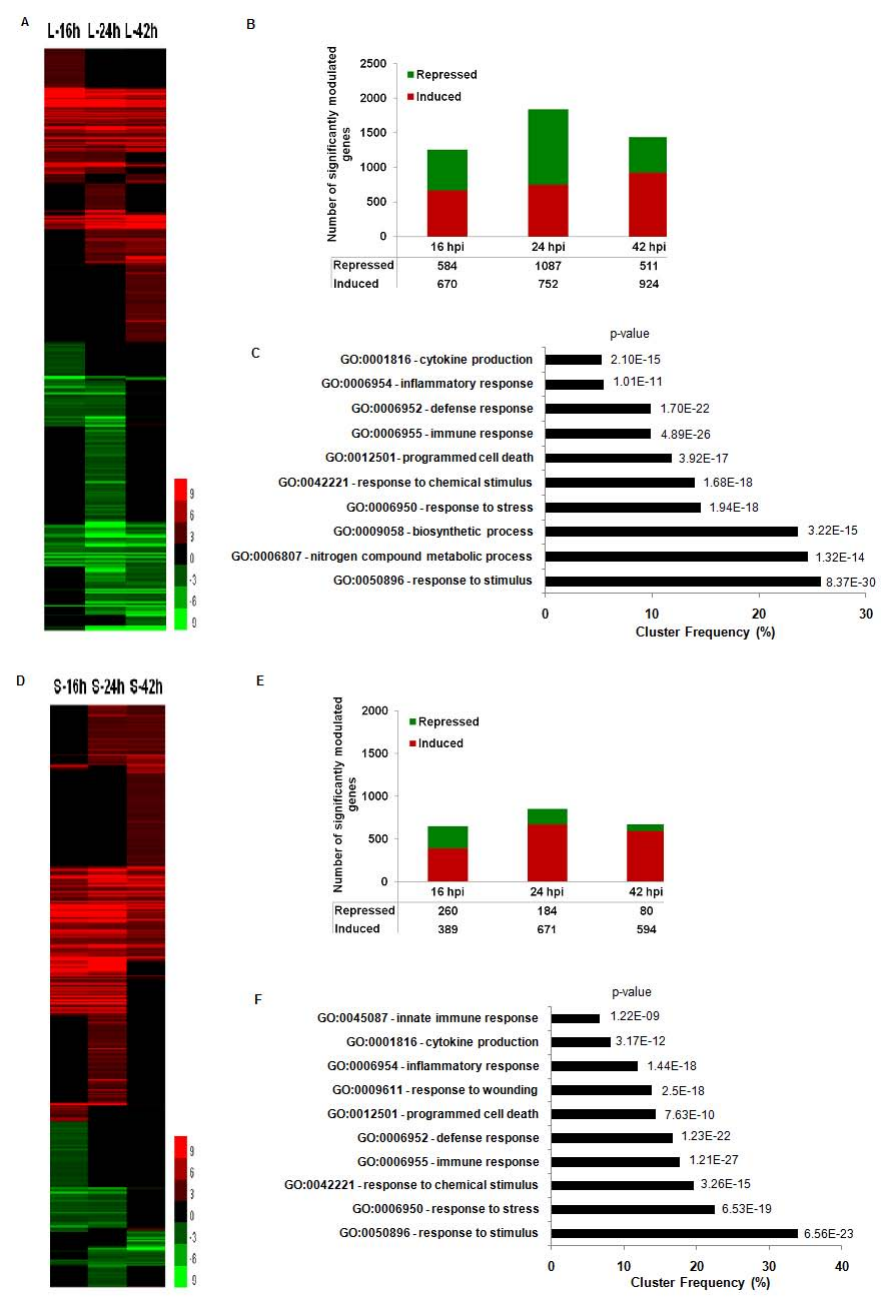
**Differential gene expression in acute *B. pseudomallei *infection over 42 hrs**. Hierarchical clustering of the expression profile of (**A**) liver (L) and (**D**) spleen (S) infected with *B. pseudomallei *for 16, 24 and 42 hpi; Number of genes modulated during acute *B. pseudomallei *D286 infection in BALB/c mice at 16 hpi, 24 hpi and 42 hpi in both (**B**) liver and (**E**) spleen; Major biological processes consistently modulated throughout the acute phase of infection in (**C**) liver and (**F**) spleen as determined by GOTerm Finder analysis.

To gain deeper insight into the host response to *B. pseudomallei *infection, we used the mouse whole-genome microarray from Illumina to elucidate the global changes of host gene expression in both infected liver and spleen. We noted that *B. pseudomallei *infection in BALB/c mice at the acute phase results in more differentially expressed genes in the liver compared to the spleen. Notably, most of the differentially expressed genes in liver at 24 hpi were down-regulated (Figure 3).

**Figure 4:**
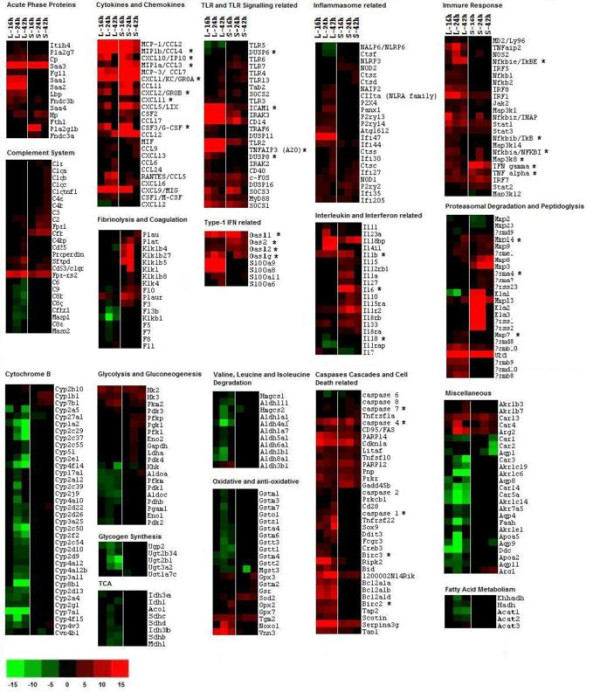
**Transcriptional responses to acute *B. pseudomallei *infection**. Hierarchical clustering of the expression profile of liver and spleen infected with *B. pseudomallei *at 16, 24 and 42 hpi according to functional categories. The heat maps indicate the fold change in liver or spleen gene expression greater than (red) or less than (green) 2-fold at least once during the time course. Genes whose expression did not change are coloured in black. *, immune-related genes known to be associated with the general bacterial infection.

In order to gain insight from the large amount of microarray data, gene expression results were analyzed in the context of biological processes utilizing GeneSpring GX7.3.1 Expression Analysis (Agilent Technologies, USA), Pathway Studio 6 (Adriane Inc.) and the web-based software GOTerm Finder and GeneTrail software. The analysis outputs consistently demonstrated that the majority of these differentially expressed genes were clustered as host immune response, defence response, cell cycle regulation, proteasomal degradation, signal transduction, and nutrient metabolism related genes (Figure 4). As expected, the early host response is enriched for immediate immune responses, including the inflammatory response, acute-phase proteins response, apoptosis and cell death programs. At 24 hpi, a majority of the genes are involved in host cellular metabolism and signal transduction pathways and found to be down-regulated. Due to the large number of significantly differentiated genes modulated during the infection, only data related to genes that have some functional information are shown and discussed below. The identified genes were categorized according to functional categories and fold change relative to naïve control mice are presented as a heatmap (Figure 4 and Additional file 2, Table S1).

### The TLR2 pathway is responsible for initiation of host defence responses to *B. pseudomallei *infection

Upon contact with the host cell, *B. pseudomallei *is known to elicit Toll-like receptor (TLR) signalling through transmembrane pattern recognition receptors (PRR) [12-14]. In this study, the expression levels of several TLRs (TLR2, 3, 4, 5, 6 and 7) were modulated; TLR2 was highly induced throughout the 42 hr time point in the liver. In contrast, TLR4, which detects lipopolysaccharides (LPS), was induced weakly at 42 hpi (Figure 4 and Additional file 2, Table S1). This expression profile is similar to that reported by Feterl et al. [12] in *B. pseudomallei*-infected RAW264.7 macrophages.

**Figure 5:**
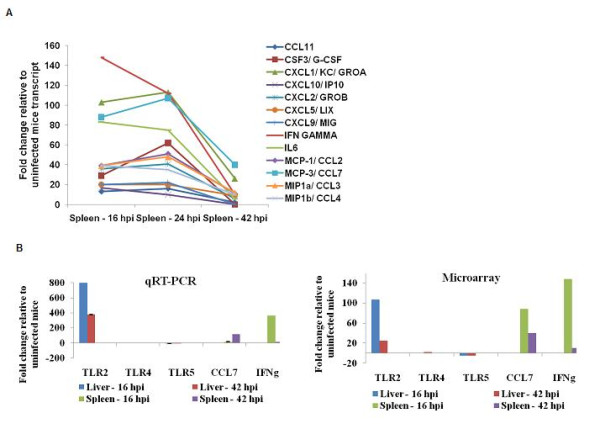
**Expression profiling of some immune-related genes over 42 hrs**. (**A**) Decrease in transcriptional expression of potent cytokines and chemokines in the spleen at 42 hpi, (**B**) qPCR analysis of host-cell genes found to be differentially expressed by microarray.

Engagement of TLRs upon *B. pseudomallei *infection subsequently altered various immune responses particularly the inflammation related genes. These include the pro-inflammatory mediators (TNF, IL1b, IL6, colony stimulating factor 3 (GCSF), colony stimulating factor 1 (MCSF)), the chemokines (CCL3, CCL4, CXCL1, CXCL2, CXCL3), and the IFN-stimulated genes (ISGs) (2'-5' oligoadenylate synthetase (OAS), the IFN-inducible chemokine genes (CCL9, CXCL10, CXCL11)). Genes that activated the immune response included the NFκB family members and their co-activator B-cell leukemia (BCL3), and the activator protein-1 components (JUNB and fos-like antigen 2 (FOSL2)), while factors that mediate the effects of IFN (interferon regulatory factor (IRF) 1, IRF4, IRF7, signal transducer and activator of transcription (STAT) 1, STAT2, STAT3) were also up-regulated in response to infection. Of note, in the spleen, many of these inflammatory genes were highly elevated at 16 hpi, peaked at 24 hpi, followed by a drastic decline at 42 hpi (Figure 5a). These include the IFNγ, the chemokines CXCL1 and CXCL2 which are important for neutrophil migration and mobilization; as well as GCSF, CXCL2, CXCL10 and IL6 (Figure 4 and Additional file 2, Table S1). The relative expression of selected differentially regulated host-cell genes was analysed by quantitative Real Time Polymerase Chain Reaction (qRT-PCR) on the same samples as those analysed by microarray analysis (Figure 5b). The samples were verified by the qRT-PCR as up- or down-regulated, albeit with magnitudes different from those recorded by the microarray analysis.

Genes that contribute to negative feedback loops that allow the cell to return to its inactivated state were also up-regulated. These include NFκBIA and NFκBIe which sequester NFκB proteins in the cytoplasm, suppressors of NFκB (TNFAIP3), TLR signalling negative regulators (suppressor of cytokine signalling (SOCS), interleukin-1 receptor-associated kinase 3 (IRAK-M)), dual specificity phosphatase (DUSP) family members (DUSP8, DUSP16) and the anti-inflammatory cytokine IL10 (Figure 4 and Additional file 2, Table S1).

### Suboptimal activation of complement cascade

Activation of the complement system is important in defending against pyrogenic bacterial infection, bridging innate and adaptive immunity, and disposing of immune complexes and the products of inflammatory injury [15,16]. In this study, the genes involved in the complement system were mildly up-regulated in both organs although dominant in the spleen after 24 hpi. These include the complement component 1 (C1r and C1q), C2, C3, C4, CFB, properdin, CD55, CD93, surfactant associated protein D (SFTPD) and formyl peptide receptor involved in C3a anaphylatoxin receptor activation (FPR) (Figure 4 and Additional file 2, Table S1). However, some key genes in the mannose-binding lectin pathway (LMAN2, mannan-binding lectin serine peptidase (MASP) 1, MASP2) and membrane-attack complex (MAC) (C6, C6a, C8b, C8g, C9, CFHR1) formation were down-regulated. A summary of the modulated genes within the complement system is shown in Additional file 3, Figure S2. Activation of complement can also be enhanced in a pathogen-independent manner by acute-phase proteins and triggered by the proteins within the coagulation or fibrinolysis pathways. The fibrinolysis related genes (plasminogen activator, urokinase, (PLAU), plasminogen activator, urokinase receptor (PLAUR), PLAT, kallikrein (KLK) 1, KLK4) were also elevated after 24 hpi in the liver. However, genes involved in the coagulation pathway (F3, F5, F7, F8, F10, F11, F13) were down-regulated at 24 hr in the liver (Figure 4 and Additional file 2, Table S1).

### Activation of caspases and cell death programs

Several Nod-like receptor (NLR) family genes (nucleotide-binding oligomerization domain (NOD) 1, NOD2, NLRP2, NLRP3 and class II trans-activator (CIITA)) which act as intracellular sensors to detect cytosolic microbial components and "danger" signals were elevated upon infection (Figure 4 and Additional file 2, Table S1). This subsequently triggered the activation of caspase (CASP) cascades to execute apoptosis and amplify the inflammatory responses essential in controlling intracellular pathogens. Various caspases, including the subfamily of inflammatory mediator (CASP1, CASP4), the apoptotic activator (CASP2, CASP8) and the apoptotic executioner (CASP7) [17-19] were up-regulated in response to infection. Furthermore, the cell death associated genes (BCL2 family members, BH3 interacting domain death agonist (BID), CD28, cyclin-dependent kinase inhibitor 1A (CDKN1A), SCOTIN, serine peptidase inhibitor, clade A (SERPINA), and anti-apoptotic factors baculoviral IAP repeat-containing (BIRC) 2 and BIRC3) were also elevated in the *B. pseudomallei*-infected host over the 42 hr time period.

Many Gram-negative bacteria, such as *Salmonella typhimurium *(*S. typhimurium*), *Pseudomonas aeruginosa *(*P. aeruginosa*), *Legionella pneumophila *(*L. pneumophila*) and *Francisella tularensis *(*F. tularensis*) can induce caspase 1 activation and rapid macrophage cell death by inflammasome activation [20-22]. The caspase 1 dependent macrophage death induced by *B. pseudomallei *reported recently by Sun et al. [23] and the induction of IL1b and IL33 were also observed in this study. Our expression profiles indicated that additional inflammasome-related genes were up regulated at 24 hpi. For example, genes encoding proteins involved in the NLRP3 inflammasome were up regulated: members of the cathepsin family (capthepsin C, D, F, S and Z), purinergic receptor family members (P2RX4, P2RY2, P2RY13, P2RY14), pannexin-1 (PANX1) and autophagy related gene (ATG16l2) (Figure 4 and Additional file 2, Table S1) [19,24]. In addition, the type 1 IFN related genes (OAS1G, OAS2, OASL1, OASL2, S100A6, S100A8, S100A9, S100A11) that are necessary for activation of the inflammasome in *Francisella novida*-infected macrophages [22], were highly induced over the course of infection and peaked at 24 hpi.

### Prolonged expression of acute phase responses may lead to tissue injury

Acute phase proteins (APP) are important in providing protective functions at sites of tissue injury [25], however their maintenance over long periods may have negative clinical consequences [26]. The APP isolate and neutralize the pathogen and prevent further pathogen entry while minimizing tissue damage and promoting repair processes, thereby permitting host homeostatic mechanisms to rapidly restore normal physiological functions [26]. Numerous APP (ceruloplasmin (CP), haptoglobin (HP), phospholipase A2 (PLA2G), serum amyloid A (SAA)) were up-regulated during the *B. pseudomallei *acute infection. Among these, family of SAA (particularly the SAA2 and SAA3) was highly induced throughout the infection period. SAA mRNA and protein synthesis are induced *in viv*o during the inflammatory response towards various challenges such as tissue damage, infection and trauma in all vertebrate species. However, prolonged expression of SAA, and the consequent long-term production of the extracellular matrix-degrading enzymes, may play a role in degenerative diseases [26]. This expression profile indicates tissue damage has occurred in the host, which can lead to induction of the ubiquitin system, peptidoglysis and proteasomal degradation. Indeed, the ubiquitin D (UBD) gene required to label proteins for proteasomal degradation, peptidoglysis associated genes (elastases, matrix metalloproteinease) as well as genes encoding the proteasome, a multi-subunit complex that degrades proteins targeted for destruction by the ubiquitin pathway, were significantly induced beginning at 16 hpi (Figure 4 and Additional file 2, Table S1).

### Suppression of various metabolic pathways alters liver cellular homeostasis

Gene expression profiles revealed a number of genes coding for various metabolic enzymes were down-regulated in the liver after 24 hpi. Gene ontology identified that most of the suppressed genes were involved in oxidation reduction, organic and carboxylic acid metabolic processes, electron carrier activity, lipid metabolic processes etc. GeneTrail analysis revealed most of these genes including acetyl-coenzyme A acyltransferase 1B (ACAA1B), acyl-coenzyme A dehydrogenase, medium chain (ACADM), acetyl-coenzyme A acetyltransferase (ACAT), aconitase 1 (ACO1), aldehyde dehydrogenase (ALDH), enolase 1 (ENO1), enoyl-coenzyme A (EHHADH), 3-hydroxy-3-methylglutaryl-coenzyme A synthase 2 (HMGCS2), code for proteins involved in the amino acid metabolism pathways. The top ten pathways suppressed following *B. pseudomallei *infection are shown in Table 1.

**Table 1:** List of major biological processes down-regulated at 24 hpi in the liver identified by GOTerm Finder

KEGG Pathways	p-value
Biosynthesis of unsaturated fatty acids	9.98E-05
Fatty acid metabolism	9.98E-05
Valine, leucine and isoleucine degradation	9.98E-05
Tryptophan metabolism	0.000141038
Butanoate metabolism	0.00571029
Drug metabolism - cytochrome P450	0.00571029
Biosynthesis of alkaloids derived from ornithine, lysine and nicotinic acid	0.0112801
Propanoate metabolism	0.0112801
Arginine and proline metabolism	0.0198866
Biosynthesis of phenylpropanoids	0.0198866

Cytochrome B has a crucial role in the activity of the *bc*_1 _complex, one of several complexes that contribute to energy transduction in the mitochondria [27]. Surprisingly, a number of cytochrome B genes associated with phosphorylation-dependent pathways (electron transport chain) and cytochrome P450 metabolism of xenobiotics were significantly down-regulated after 24 hpi (Figure 4 and Additional file 2, Table S1).

Many enzymes associated with essential pathways are modulated during *B. pseudomallei *acute infection. Glycolysis is a central pathway that produces important precursor metabolites including glucose-6-phosphate and pyruvate. Many of the glycolytic enzymes were significantly down-regulated, including phosphofructokinase (PFK1), PFKP, aldolase 1, A isoform (ALDOA), ALDOC, phosphoglycerate mutase 1 (PGAM1), ENO1, ENO2, as well as pyruvate dehydrogenase beta (PDHB), the key enzyme that converts pyruvate to acetyl-CoA for energy production via the TCA (tricarboxylic acid) cycle (Figure 4 and Additional file 2, Table S1). A number of genes encoding enzymes involved in the TCA cycle were also down-regulated. In addition, the alternative pathways involved in producing acetyl-CoA or TCA cycle components such as the fatty acid metabolism, tyrosine metabolism as well as valine, leucine and isoleucine degradation pathways, were also down-regulated. The modulation profile of glycolysis and TCA cycle in response to *B. pseudomallei *acute infection is summarized in Additional file 4, Figure S3.

## Discussion

Individuals with acute melioidosis present symptoms rapidly and succumb to disease (<24 hpi) before antibiotic treatment can be administrated [1]. Previous studies on elucidating the pathogen-host response of melioidosis had focused primarily on a subset of immune response genes [8,9,28-30], however, analyses of single gene or limited gene expression patterns is insufficient to dissect the host response to infection globally. We developed an acute melioidosis model in BALB/c mice to get a comprehensive genome wide view of the host transcriptional response during the acute stage of melioidosis. Our analyses clearly demonstrated that the pathogen had intimately engaged the innate immune system at the early onset of infection by rapid induction of numerous inflammatory responses.

The primary response observed was the overwhelming induction of TLR2 to counteract *B. pseudomallei*, which we propose, subsequently triggered the activation of many inflammation-biased genes important in attracting neutrophils and monocytes to the site of acute inflammation. These cytokines and chemokines also function as central mediators in activating various host defence systems such as apoptosis, JAK/STAT signalling pathway, mitogen activated protein kinase (MAPK) signalling pathway and ultimately trigger the appropriate adaptive immune system. Induction of these genes was previously reported in numerous *in-vivo*, *in-vitro *or melioidosis patient studies [2,13,28,30-32]. Hence our study reinforces the consistency of the inflammatory genes expression in response to acute melioidosis. Concomitantly, the host frontline defence system is boosted by increasing the production of granulocytes (Figure 2). Nevertheless, the bacteria are capable of propagating in a tissue environment that is evidently overloaded with high levels of inflammatory-associated proteins (Figure 1). This genome-wide expression study confirms that the production of signals responsible for the activation of pro-inflammatory genes in response to *B. pseudomallei *infection, are mainly TLR2 dependent. This observation supports a previous finding of improved survival in respiratory infection in TLR2 KO mice with reduced bacterial burden and lung inflammation, as well as less distant organ injury [13].

The cluster of inflammatory-associated genes consistently highly induced in response to *B. pseudomallei *acute infection is part of the group designated as "common host immune response". Most of these genes are induced in many different cell types in response to exposure to several different pathogen species such as *Escherichia coli*, *Salmonella typhi*, *Staphylococcus aureus*, *Listeria monocytogenes*, *Mycobacterium tuberculosis*, *Candida albicans*, *Bordetella pertussis*, *Mycobacterium bovis, P. aeruginosa *and *S. typhimurium *[33-39]. Up-regulation of this core set of genes by pathogens might represent a general "alarm signal" for inflammatory infections [39]. Common host genes (such as CCL2, CCL7, IP30 and genes encoding MHC class II related molecules) known to be repressed by pathogens have been identified in PBMCs infected with *B. pertussis*, *E. coli *and *S. aureus *[36]. Surprisingly in our study, these genes were highly induced in response to *B. pseudomallei *infection and could be a *Burkholderia *specific response.

The reaction to a given pathogen must be sufficient for bacterial elimination but not so strong as to be harmful to the host [40]. This is particularly true for innate immunity in cases including acute melioidosis where excessive activation of inflammatory genes is commonly associated with septic shock. We did not see up-regulation in the levels of anti-inflammatory signals and TLR negative regulators at 24 hpi, suggesting that the failure to suppress inflammation at this early time point contributes to the excessive inflammation and acute nature of this infection. Nevertheless, at 42 hpi, a significant decrease in expression of these potent inflammatory genes (Figure 5a) was observed and may actually benefit the intracellular pathogen. However, the underlying factors that contribute to the decrease in expression of these inflammatory genes remain unclear as the production of anti-inflammatory cytokines (IL4, IL6 and IL10) was relatively insufficient to counter the high pro-inflammatory responses at 24 hpi.

Acute forms of melioidosis that lead to sepsis, multiple organ failure and death are thought to result from an uncontrolled inflammatory reaction that ultimately leads to excessive inflammation [7] and eventually tissue injury in the *B. pseudomallei*-infected host. Activation of proteasomal degradation following tissue injury suggests the production of immunological waste products such as apoptotic cells and immune complexes in the *B. pseudomallei*-infected host. This could be attributed to a failure in activating the complement system in time, leading to the accumulation of waste and uncontrolled spread of the pathogen (Figure 1). The low levels of the potent anaphyatoxin C5a observed in our study most likely inhibit the downstream terminal complement pathway. As a result, deficient rapid clearance of apoptotic cells resulting in extracellular disintegration of the cell and release of intracellular components triggers inflammatory cytokine production and contributes to "breaking tolerance" by facilitating an immune response to intracellular constituents [41]. This is the first evidence of failure of the downstream complement pathway in acute melioidosis.

The *B. pseudomallei*-infected host also over express many cell death related genes which suggests that the host initiates various cell death defence responses and disrupts cell regulation to limit a favourable intracellular niche for the pathogens. Elevation of caspase 2, 3, 7 and 8, as well as the BCL-2 family protein BID and TNF-receptor superfamily suggests that the host triggers apoptosis signalling via the death receptor mediated (extrinsic) pathway. In addition, we saw an up regulation of inflammasome related genes (NAIP2, NLRP3, CIITA, NLRP6, interferon activated gene 205 (IFI205)) not previously reported in the *B. pseudomallei*-infected host. *B. pseudomallei *virulence factors such as type-three secretion factors (TTSS), flagellin and channel forming toxins like hemolysin could trigger inflammasome-dependent caspase 1 activation [6,42].

*B. pseudomallei *is known to interfere with iNOS expression in RAW264.7 macrophages and abrogate nitric oxide (NO) production during the early stages of infection [12,43]. Arginase 1 and arginase 2 have been reported to compete with NO syntheses for their common substrate, arginine, and prevent NO production in the *M. tuberculosis *infected bone-marrow derived macrophages as well as *Salmonella *infected RAW264.7 macrophages [44-46]. Here we report for the first time that *B. pseudomallei *up-regulates both arginase 1 and arginase 2 isoforms in the host with arginase 2 being more dominant. The expression profiles demonstrate both host nitric oxide synthase 2 (NOS2) and arginase 2 were elevated at a similar magnitude at 24 hpi. This suggests that arginase competes with NOS2 to produce NO from arginine during the infection, leading to the suboptimal antibacterial effect of NOS2 in the *B. pseudomallei*-infected host.

Certain pathogens evade the host defence by triggering the TLR2-mediated biased anti-inflammatory effects or prevent recognition by TLRs [47]. For example, Yersinia- and Candida-induced TLR2 signalling leads to the release of IL-10, which can lead to immunosuppression. However, the response following recognition of *B. pseudomallei *via the TLR2 signalling pathway is contrary to the evasion mechanism exploited by *Yersina *spp. and *Candida *spp. In addition, some pathogens have developed strategies to either block or avoid their recognition by TLRs and subsequent activation of the innate defence. This study suggests that *B. pseudomallei *may use specific TLR-mediated signals to escape from the host defence. Future studies will be aimed at determining whether *B. pseudomallei *utilizes these signals to evade TLR clearance mechanisms.

Tissue injury leads to extracellular matrix breakdown, including the degradation of hyaluronic acid (HA) and resulting oligosaccharides. In this study, the gene encoding hyaluronan synthase 2 (HAS2), the enzyme that produces HA, was induced. In contrast, the genes encoding hyaluronoglucosaminidases (HYAL1, HYAL2), the enzymes that degrade HA, were repressed, indicating that perhaps HA is not degraded during a *B. pseudomallei *infection. These endogenous signals can also trigger TLR2 and/or TLR4 activation and signals distinct from microbial stimulators, for instance HA but not LPS, signal through TLR4, MD2, and CD44 [48]. Up regulation of TLR2, TLR4 and TLR7 as well as MD2 could indicate *B. pseudomallei*-infected host responses to endogenous signals released during tissue damage [48]. However, the ability of the engaged TLRs to distinguish between microbial and endogenous signals and subsequently trigger appropriate responses, remains unclear [40,48,49]. These observations reflect that the inflammatory response may cause more damage to the host than the microbe. In summary, our work has provided an extensive description of host defence responses to *B. pseudomallei *during an acute infection.

Changes in host cell metabolism as a consequence of nutrient scavenging by intracellular *B. pseudomallei *have never been studied. The microarray data presented here provides the first description of changes in the *B. pseudomallei*-infected host cell metabolism particularly the glycolytic and TCA pathways. The glycolysis pathway and the TCA cycle were both transcriptionally repressed. It remains to be determined if shutting down both these pathways is part of the host response to control the replication of intracellular bacteria or a strategy adopted by the pathogen to survive intracellularly. In addition, we found that expression of 37 cytochrome P450-related genes was suppressed in the liver over the course of infection, most notably at 24 hpi. The expression of the detoxification enzymes amine UDP-glucuronosyltransferases (UGT2B1, UGT2B34) and N-sulfotransferase (NDST1) was also down-regulated. Our data suggests that *B. pseudomallei-*induced impaired liver detoxifying activity might be a causative factor in liver sepsis. Collectively, the data presented here suggests that hepatocytes, via receptors for many pro-inflammatory cytokines, modify their metabolic pathways (glycolysis, TCA, fatty acid metabolism and various amino acid biosynthesis) in response to *B. pseudomallei *acute infection.

## Conclusion

This genome wide expression profile demonstrates that a general "alarm signal" of infection is triggered by the host upon infection with *B. pseudomallei *and subsequently various defence programs are activated to control the replication of the intracellular pathogen. Nevertheless, the overwhelmed inflammatory response to infection as well as tissue injury leads to metabolic disturbances and homeostatic imbalance which is detrimental to the host. The suboptimal complement function correlates with uncontrolled spread of the bacteria, a hallmark of the acute nature of this infection. In addition, we postulate tissue damage following *B. pseudomallei *acute infection is contributing to dysregulation of the innate immune response via TLR2, the surveillance receptor that recognizes both endogenous and exogenous molecules.

## Methods

### Animals

7- to 9-week-old BALB/c mice were purchased from the Institute for Medical Research, Malaysia. They were housed in High Temperature Polysufone (Techniplast, Italy) cages with a bedding of wood shavings, subjected to a 12 hr light/dark cycle and fed on a diet of commercial pellets and distilled water ad libitum. All animal experiments were performed in accordance with the Universiti Kebangsaan Malaysia animal ethics guidelines and approved by the Universiti Kebangsaan Malaysia Animal Ethics Committee (UKMAEC).

### Bacteria

The three clinical *B. pseudomallei *isolates (referred to herein as D286, R15 and H10) used in this study are listed in Table 2. All *B. pseudomallei *isolates were previously characterized based on biochemical tests as well as by 16 S rRNA sequencing [50]. Genome comparison with *B. pseudomallei *strain K96243 and *B. thailandensis *strain E264 identified *B. pseudomallei *D286, R15 and H10 as members of the YLF genomic group (Lye et al., unpublished data). Bacteria were grown in Brain Heart Infusion (BHI) broth overnight at 37°C. The cells were centrifuged at 10,000 × g, suspended in BHI broth containing 20% glycerol, frozen immediately in aliquots of 10^9 ^CFU per ml and stored at -80°C [5].

**Table 2:** Description of *B. pseudomallei *isolates utilized in this study

Isolate	Source	Year	Reference
Human D286	Kuala Lumpur Hospital, Malaysia	1986	[50]
Human R15	Institute for Medical Research, Kuala Lumpur, Malaysia	2005	[50]
Human H10	Raub General Hospital, Pahang, Malaysia	1995	[50]

### Determination of 50% lethal dose (LD_50_)

Mice were divided into four groups of five BALB/c mice and each group was inoculated with a bacterial isolate at different doses via the lateral tail vein. The doses reported reflect the actual dose of the inoculums as determined by colony counts on Ashdown agar. Five control mice received 200 μl of sterile phosphate-buffered saline (PBS). Following inoculation, mice were monitored daily over 10 days for signs of morbidity and mortality [11,51,52].

### Enumeration of viable *B. pseudomallei *in the blood

Mice were tail-bled on days 2, 4, 6, and 8 post-infection. Blood was pooled for each group of mice and collected in EDTA-tubes. The blood was then plated on Ashdown agar and colonies were counted after 2 days incubation at 37°C.

### Infection of mice and preparation of organs

Infection experiments were performed as described previously with minor modification [5,11]. In brief, for each infection, an aliquot of the freshly thawed *B. pseudomallei *D286 suspension was adjusted to a density equivalent to that of a no. 0.5 McFarland nephelometer standard (approximately 1.5 × 10^8 ^CFU/ml). The suspension was then diluted to the appropriate concentration (10 × LD_50_) in sterile PBS for inoculation into mice as described previously [5]. A bacterial suspension of 0.2 ml was injected into the lateral tail vein. The actual number of administered bacteria was determined for each experiment by plating on Ashdown agar and counting CFU after 48 hr. At 16, 24, and 42 hpi, three infected mice were euthanized by ether inhalation to determine the number of CFU present in blood, liver and spleen. Liver and spleen were aseptically removed and homogenized in 2 ml of sterile PBS using a handheld motorized homogeniser (IKA^® ^T10 Basic Ultra-Turrax^®^, Germany). Organ homogenates were serially diluted ten-fold with PBS and 100 μl of each dilution was plated on Ashdown agar. The number of bacteria was counted as CFU per organ. For the determination of blood CFU, an undiluted 0.1 ml sample collected in EDTA-tubes was plated out and the number of CFU/ml was determined. At each time point, a further 3 infected mice were euthanized for immediate RNA isolation.

### Leukocyte differential counts

To determine the leukocyte differential counts, blood from infected mice were used to make a smear. The slides were fixed in 100% methanol and stained with Wright's and Giemsa stains (Sigma, USA) according to the manufacturer's instructions.

### Gene expression analyses

Microarray experiments were performed using the SentrixMouseRef-8 Expression BeadChips (Illumina, USA), containing over 24000 probes according to the instructions provided. Three biological replicates were performed for each sample from each time point. The organ samples were homogenized using a handheld motorized homogeniser (IKA^® ^T10 Basic Ultra-Turrax^®^, Germany). Total RNA was extracted using TRIzol (Invitrogen, USA), DNase treated and RNA purified by Qiagen kits (Qiagen, Germany) according to the manufacturers' instructions. The RNA integrity and concentration was assessed on the Agilent 2100 Bioanalyzer (Agilent Technologies) and RNA 6000 LabChip^® ^kit as well as the Nanodrop ND-1000 spectrophotometer (Agilent Technologies).

Total RNA (350 ng) from each sample was reverse transcribed to cDNA and in vitro transcription of cDNA to cRNA was performed overnight (14 hr) using Ambion's Illumina RNA Amplification kit according to the manufacturer's instructions (Ambion, USA). The cRNA concentrations and integrity were assessed as above. Labelled cRNA (750 ng) was hybridized overnight (17 hr) to the Illumina Sentrix MouseRef-8 expression BeadChip array V1.1 (Illumina, USA), and arrays were washed, blocked, stained and scanned on an Illumina BeadArray Reader following the manufacturer's protocols as previously described [53,54] with some modifications.

### Microarray data analysis

The BeadStudio version 1.0 (Illumina, USA) software was used to generate signal intensity values from the scans. After that, the standard normalization procedure for one-colour array data in GeneSpring GX7.3.1 Expression Analysis (Agilent Technologies, USA) was used. In brief, data transformation was corrected for low signal, with intensity values <10 set to 10. In addition, per-gene normalization was applied by dividing each probe intensity by the median intensity value for all samples. The normalized data were grouped on the basis of the experimental conditions (organs and infection time points) and filtered using the Volcano Plot. Differentially expressed genes were defined as those having a *p *value of ≤ 0.05 and an absolute change greater than 2-fold for *B. pseudomallei *infected tissue at 16 hr, 24 hr or 42 hr relative to the uninfected control tissue. The data discussed in this publication have been deposited in the NCBI Gene Expression Omnibus and are accessible through the GEO Series accession number GSE25074 http://www.ncbi.nlm.nih.gov/geo/query/acc.cgi?acc=GSE25074.

The identified differentially expressed gene lists from each experimental condition were compared in a Venn diagram using the web-based software VENNY http://bioinfogp.cnb.csic.es/tools/venny/index.html. The web-based software GOTerm Finder http://go.princeton.edu/cgi-bin/GOTermFinder and GeneTrail http://genetrail.bioinf.uni-sb.de/ were used to identify Gene Ontology (GO) and Kyoto Encyclopedia of Genes and Genomes (KEGG) categories found in specified subsets of the data. The analyses were performed by using the default setting in both software with a significance threshold p-value < 0.05.

Selected data were organized by a hierarchical clustering with the web-based software Cluster 3.0. The clustering algorithm is based on an uncentered correlation metric, with average linkage clustering and visualized using Java Treeview V1.1.3.

### Quantitative Real Time PCR

qRT-PCR was performed in the Mastercycler^® ^ep realplex (Eppendorf, Germany) to quantify the expression of *TLR2*, *TLR4*, *TLR5*, *IFNγ *and *CCL7 *genes. Briefly, 25 μl reactions were performed using the iScript™One-Step RT-PCR kit with SYBR green according to the manufacturer's instruction (BioRad Laboratories, USA), primers at a final concentration of 1 μM and a data acquisition temperature of 76°C. In order to control for variation in RNA concentration, cycle threshold (Ct) values were normalized to β-actin that does not change with infection [12]. Primer sets used in this study are shown in Additional file 5, Table S2.

## Authors' contributions

CYC conceived and performed the experiments, analyzed data and drafted the manuscript. DM and SN participated in the experimental design, data analysis and manuscript preparation. All the authors have read and approved the final manuscript.

## Supplementary Material

Additional file 1 **Figure S1 - Mortality of mice (n = 3-5 mice/group) infected intravenously with *B. pseudomallei *strain (A) D286, (B) H10 and (C) R15**. Mice were infected intravenously with doses from 10^2 ^to 10^6 ^CFU. Animals were observed daily up to ten-days, and the percentage survival plotted against time. Click here for file

Additional file 2 **Table S1 - Kinetic profiles of host gene expression modulated by *B. pseudomallei *infection in both liver and spleen**. Click here for file

Additional file 3 **Figure S2 - Transcriptional changes of genes involved in the complement system**. Shown is the expression profile for genes modulated at 42 hpi in liver and spleen. Induced genes are highlighted in red while the repressed genes are highlighted in green. Click here for file

Additional file 4 **Figure S3 - Transcriptional changes of genes involved in the glycolysis and TCA pathways**. Shown are the expression profiles for liver genes (24 hpi) encoding enzymes involved in glycolysis (left) and TCA cycle (right). Induced genes are highlighted in red while the repressed genes are highlighted in green. Click here for file

Additional file 5 **Table S2 - Primers used in qRT-PCR**. Click here for file
