# Supplementary figures and images for: Genome wide transcriptome profiling of a murine acute melioidosis model reveals new insights into how Burkholderia pseudomallei overcomes host innate immunity
Source: BMC Genomics. 2010 Nov 27;11:672. doi: 10.1186/1471-2164-11-672 (PMC3017868; doi:10.1186/1471-2164-11-672)

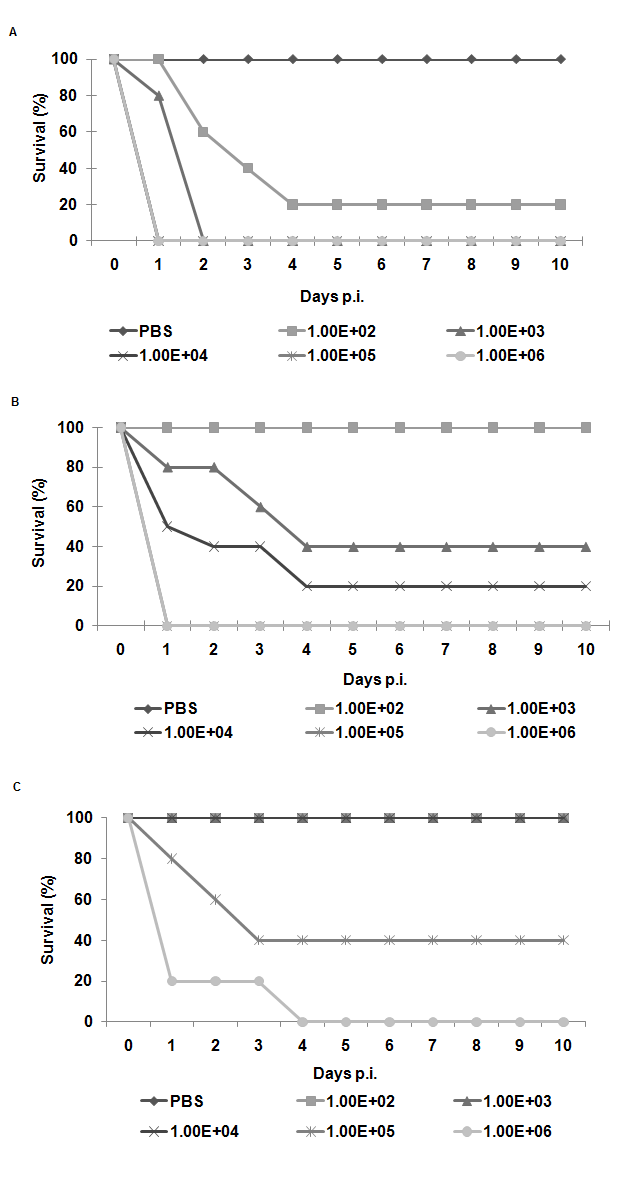

Supplement: Additional file 1 — Figure S1 - Mortality of mice (n = 3-5 mice/group) infected intravenously with B. pseudomallei strain (A) D286, (B) H10 and (C) R15. Mice were infected intravenously with doses from 102 to 106 CFU. Animals were observed daily up to ten-days, and the percentage survival plotted against time. [file 1471-2164-11-672-S1.BMP]

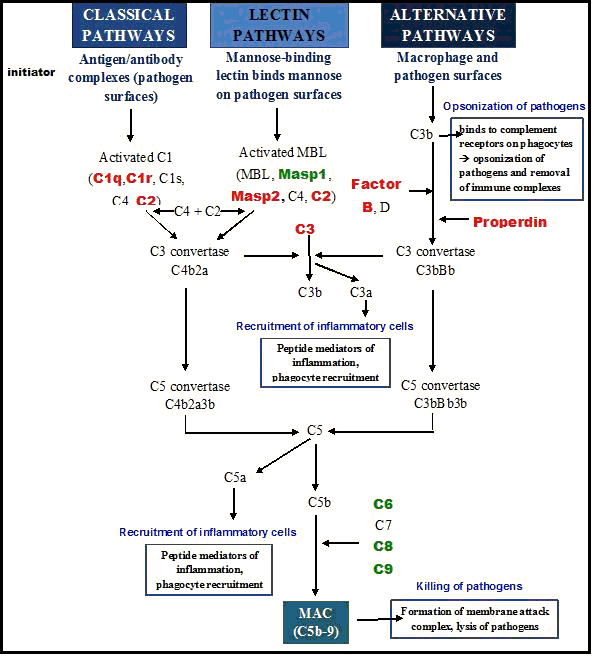

Supplement: Additional file 3 — Figure S2 - Transcriptional changes of genes involved in the complement system. Shown is the expression profile for genes modulated at 42 hpi in liver and spleen. Induced genes are highlighted in red while the repressed genes are highlighted in green. [file 1471-2164-11-672-S3.BMP]

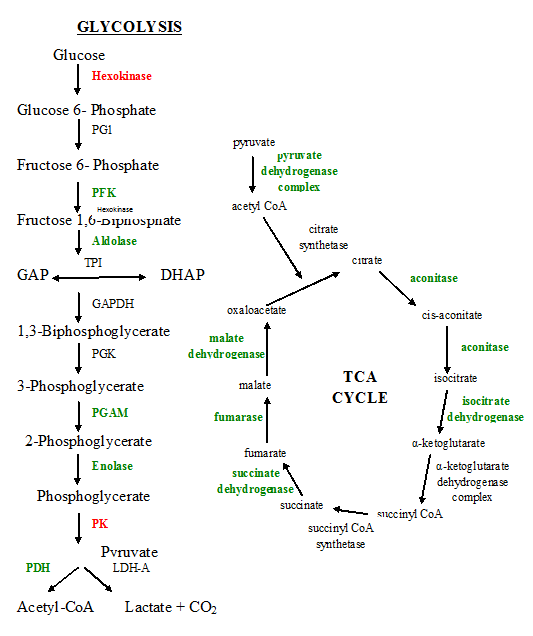

Supplement: Additional file 4 — Figure S3 - Transcriptional changes of genes involved in the glycolysis and TCA pathways. Shown are the expression profiles for liver genes (24 hpi) encoding enzymes involved in glycolysis (left) and TCA cycle (right). Induced genes are highlighted in red while the repressed genes are highlighted in green. [file 1471-2164-11-672-S4.BMP]
